# Winter behavior of Saimaa ringed seals: Non-overlapping core areas as indicators of avoidance in breeding females

**DOI:** 10.1371/journal.pone.0210266

**Published:** 2019-01-04

**Authors:** Marja Niemi, Lauri Liukkonen, Meeri Koivuniemi, Miina Auttila, Anni Rautio, Mervi Kunnasranta

**Affiliations:** 1 University of Eastern Finland, Department of Environmental and Biological Sciences, Joensuu, Finland; 2 Metsähallitus, Parks & Wildlife Finland, Savonlinna, Finland; 3 Natural Resources Institute Finland, Joensuu, Finland; University of Hong Kong, HONG KONG

## Abstract

Climate change, together with increasing human activity, poses a threat to the breeding success of endangered landlocked ringed seals (*Phoca hispida saimensis*). In this study, we estimated the spatial ecology of Saimaa ringed seals during the breeding season in the ice-covered period of December-April. The telemetry data on tagged seals (*n* = 20), with a total of 25 separate tracking periods and birth lair locations (*n* = 59) of non-tagged seals, were studied to estimate the movement ecology and breeding density. The movements of the ringed seals were more restricted during the ice-covered season; the total home range size (average 7.4 km^2^) in winter was 13 times smaller than that in summer. Individual tagged seals occupied an average of 5 ± 3 SD subnivean haul outs (snow lairs or ice cavities), and the mean distance between the haul outs was 1.6 ± 1.1 SD km (range 0.2–5.9 km). Moreover, our data indicated that ringed seal females likely exhibited breeding time avoidance of each other’s core areas, which may indicate some degree of territoriality. This was supported by the findings that the core areas (mean 1.2 km^2^) of tagged adult females (*n* = 9), did not overlap with each other. Also data on non-tagged seals showed that females did not give birth to pups within the core area radius of other parturient females. This study, together with earlier findings on the home ranges of nursed pups and perinatal mortality rates, has implications into land usage planning in Lake Saimaa by highlighting the need of undisturbed area between seal lairs and anthropogenic disturbances.

## Introduction

The life history and global distribution of ringed seals (*Phoca hispida*) are tied closely to land-fast or heavy pack ice, where the seals maintain breathing holes. When sufficient snow accumulates to form hummocks or pressure ridges, the seals dig subnivean lairs in the snow, covering the breathing holes [1, 2]. Similarly, the freshwater subspecies (*P*. *h*. *saimensis* and *P*. *h*. *ladogensis*) construct their lairs in the snowdrifts formed on the shorelines of islands and islets [3–5]. The lairs protect the ringed seals from predation and cold by providing a sheltered platform for resting (haul out lairs) and giving birth as well as nursing (birth lairs). A female gives birth to a single pup in a subnivean lair and nurses it for 6–8 weeks in the Arctic [2, 6] and 7–12 weeks in freshwater lakes [7, 8]. In general, the nursing time of the ringed seal is among the longest of the phocid seals [9].

Ringed seals are regarded as a sedentary species (e.g., [10]) but are capable of long distance movements of over 1,000 km in the Arctic. These long distance movements are typical for the open water season and are related to sea-ice edge movements and post-molting feeding excursions [11–15]. In the ice-covered season, the adult ringed seals are known to be more localized [12, 13, 16, 17]. In the lacustrine habitat, the movements of the ringed seals are suggested to be more constricted year-round [18–21]. In addition, ringed seals exhibit interannual site fidelity for their breeding areas [17, 22]; the strongest evidence of fidelity has been discovered in Lake Saimaa [3, 23], where the females exhibit an especially high degree of philopatry [24]. The ringed seal males are known to be slightly polygynous and show aggressive behavior towards other males [25], but very little is known about the female behavior in winter.

The ringed seal is one of the northern seals most vulnerable to the declines in the extent or quality of ice and snow cover [26–29]. Premature ice break-up and early lair collapse leave pups subject to increased predation, human disturbances and exposure to extreme weather conditions [30–33]. Climate change has negative effects in the endangered Saimaa ringed seal population, especially on juvenile survival [34, 35], which is already weakened due to incidental by-catch mortality [36–38]. To date, fishing restrictions have been established (e.g., [8]), and the negative effects of mild winters have been mitigated by a unique method in which volunteers pile snow along the shorelines, and the ringed seals adopt these drifts as their breeding and haul out sites [39]. Recent findings on the negative effects of land usage and other human activities on the Saimaa ringed seal population [40] suggest urgent spatial conservation needs during the breeding season, when the ringed seal is suggested to be the most sensitive to disturbance.

The aim of this study was to estimate the movement ecology of the Saimaa ringed seal in winter, with a special focus on the females. The knowledge of movement during the ice-covered period is particularly important because the current population estimates are based on the number and distribution of observed snow lairs [4, 37]. Therefore, the findings of this study can have direct implications for the conservation and monitoring of the Saimaa ringed seal in the changing climate.

## Materials and methods

Lake Saimaa, which is situated in southeastern Finland (61° 05’ to 62° 36’ N, 27° 15’ to 30° 00’ E), includes several interconnected water basins and is inhabited by a ringed seal subspecies with a current population of around 400 individuals [41]. The lake (4,400 km^2^) is approximately 180 km in length and 140 km in width with a mean depth of 12 m and a maximum depth of 85 m [42].

Between 1999 and 2017, tagged Saimaa ringed seals (10 males and 10 females) were tracked from late spring to the following winter, covering at least one month of the ice-covered season (Table 1). Four individuals had multiple interannual tracking periods. Individual annual results were taken into account, creating a total of 25 tracking data sets (Tables 1 and 2) that were treated as repeated measurements in the statistical analyses. The seals were captured at the end of the molting season in late May–early June (see [21]) in central Lake Saimaa. Overall, 16 VHF radio tags (ATS, USA), seven GPS phone tags (SMRU, UK) and two GPS-Argos satellite tags (SPLASH 10-F-297A, Wildlife Computers, USA) were attached by two-component epoxy glue to the seals’ dorsal pelage. The units were positioned with the antennae pointing backward (except for OL10 during the season of 2010–11, Table 1) for protection during ice conditions. The seals studied during each tracking period (*n* = 25) were categorized as subadults (*n* = 3, body length <100 cm and mass <40 kg) and adults (*n* = 22, Table 1). Ringed seals lose weight during the spring due to reproductive and molting activities [33, 43], and the length measurements of living individuals in field conditions are often inaccurate. Therefore, other age measurement criteria, such as known breeding or the earlier identified as an adult verified by photo-identification data [44], were also used for some of the smaller individuals, which were consequently classified as adults, although their lengths or weights did not fully fit the adult seal criteria [33]. After tagging, seals were released in the vicinity of their capture sites. Capture and handling methods were reviewed and approved by the local environmental authority Centre for Economic Development, Transport and the Environment (ESAELY/433/07.01/2012 and ESA-2008-L-519-254) and the Animal Experiment Board in Finland (ESAVI/8269/04.10.07/2013 and ESAVI-2010-08380/Ym-23) and their regulations were followed. The research was partly conducted in the Linnansaari National Park and carried out under the state-owned enterprise Metsähallitus research permit (921/662/2006). Metsähallitus is responsible for the management of one third of Finland’s surface area. No other permits were needed to conduct the research.

**Table 1:** Description of the tagged Saimaa ringed seals.

Seal ID	Age/ Sex	Kg	Body length (cm)*	Tracking method	Total tracking season dd/mo/yy					Ice-covered tracking season dd/mo/yy						Ice cover		Ice melt
					Start	End	*n* Fixes	Days	*n* Fixes/d	Start	End	*n* Fixes	Days	*n* Fixes/d				
MI99	Ad F	66		VHF	25/05/99	14/04/00	70	325	0.2	21/12/99	14/04/00	34	115	0.3	08/12/99		30/04/00
UR99	Sub M	37		VHF	26/05/99	28/03/00	54	307	0.2	30/12/99	28/03/00	18	89	0.2	08/12/99		30/04/00
	Ad M	60	126	VHF	30/05/06	29/04/07	119	334	0.4	31/01/07	24/04/07	43	83	0.5	12/01/07		24/04/07
	Ad M	60	103	VHF	27/05/07	03/05/08	87	342	0.3	05/01/08	16/04/08	28	102	0.3	02/01/08		02/05/08
EL06	Ad F	58	104	VHF	21/05/06	20/03/07	115	303	0.4	17/01/07	20/03/07	31	62	0.5	12/01/07		24/04/07
HE07	Ad F	57	111	VHF	01/06/07	03/05/08	86	337	0.3	02/01/08	15/04/08	37	104	0.4	02/01/08		02/05/08
	Ad F	58	125	VHF	22/05/11	11/04/12	135	325	0.4	22/01/12	11/04/12	39	80	0.5	02/01/12		07/05/12
KJ07	Ad M	52	110	VHF	25/05/07	03/05/08	72	344	0.2	02/01/08	16/04/08	20	105	0.2	02/01/08		02/05/08
TO07	Ad M	55	113	GPS/GSM	03/06/09	30/03/10	3860	300	12.9	14/12/09	28/03/10	201	104	1.9	14/12/09		04/05/10
PA09	Sub M	27	90	VHF	22/05/09	31/03/10	117	313	0.4	22/12/09	31/03/10	16	99	0.2	14/12/09		04/05/10
KA10	Ad F	42	115	VHF	21/05/10	27/02/11	104	282	0.4	08/12/10	27/02/11	27	81	0.3	27/11/10		08/05/11
NO10	Sub F	32	99	VHF	25/05/10	05/04/11	138	315	0.4	02/12/10	05/04/11	60	124	0.5	27/11/10		08/05/11
OL10	Ad F	59	126	GPS/GSM	31/05/10	02/04/11	2793	306	9.1	27/11/10	02/04/11	638	126	5.1	27/11/10		08/05/11
	Ad F	60	132	VHF	21/05/11	05/04/12	131	320	0.4	16/01/12	05/04/12	37	80	0.5	02/01/12		07/05/12
LI10	Ad F	48	111	VHF	21/05/10	01/04/11	109	315	0.3	02/12/10	01/04/11	54	120	0.5	27/11/10		08/05/11
TE07	Ad F	40	114	VHF	27/05/07	09/09/08	147	471	0.3	04/02/08	02/05/08	38	88	0.4	02/01/08		02/05/08
	Ad F	52	113	GPS/GSM	31/05/11	13/02/12	6424	258	24.9	02/01/12	13/02/12	122	42	2.9	02/01/12		07/05/12
AS12	Ad M	57	135	GPS/GSM	20/05/12	09/01/13	5417	234	23.1	02/12/12	09/01/13	239	38	6.3	02/12/12		05/05/13
VO12	Ad M	63	130	GPS/GSM	28/05/12	02/04/13	2045	309	6.6	02/12/12	02/04/13	83	121	0.7	02/12/12		05/05/13
HH12	Ad F	42	109	VHF	24/05/12	01/03/13	83	281	0.3	07/12/12	01/03/13	25	84	0.3	02/12/12		05/05/13
NI09	Ad M	59	129	VHF	31/05/12	02/04/13	118	306	0.4	08/12/12	02/04/13	37	115	0.3	02/12/12		05/05/13
MI13	Ad M	57	133	GPS/GSM	03/06/13	26/04/14	2277	327	7.0	11/01/14	12/04/14	107	91	1.2	11/01/14		13/04/14
JE14	Ad M	56	124	GPS/GSM	27/05/14	08/02/15	3891	257	15.1	13/01/15	08/02/15	30	26	1.2	11/01/15		08/04/15
PA16	Ad M	50	114	GPS/Argos	27/05/16	26/02/17	288	275	1.0	10/01/17	26/02/17	22	47	0.5	04/01/17		24/04/17
PI16	Ad F	47	115	GPS/Argos	29/05/16	28/02/17	530	275	1.9	15/01/17	28/02/17	55	44	1.3	04/01/17		24/04/17
Average		52	117				1168	310	4.3			82	87	1.1			

Individuals with multiple tracking seasons are shown in light gray. Ad = adult, Sub = subadult, F = female, M = male, d = day.

*Nose-tail measure.

**Table 2:** Saimaa ringed seal winter season home ranges (mean ± SD, km^2^), number of haul out sites, and the portion (%) of haul out sites confirmed to have lair structures. The data consist of 20 tagged seals, from which four had multiple interannual tracking periods, making the total *n* 25.

	*n*	MCP100	MCP50	Haul out sites (% were found to be lairs)	Haul out sites (lair % of found lairs) in core area
Adult F	12	4.5 ± 2.9	1.2 ± 1.1	4.5 ± 2.1 (43%)	2.6 ± 1.3 (68%)
Adult M	10	11.5 ± 7.3	2.1 ± 2.0	5.0 ± 3.2 (14%)	2.1 ± 1.4 (86%)
Subadult	3	5.2 ± 4.6	1.9 ± 2.4	3.7 ± 3.1 (46%)	1.7 ± 1.2 (60%)
Total	25	7.4 ± 6.1	1.7 ± 1.7	4.5 ± 2.6 (30%)	2.2 ± 1.3 (71%)

Lake Saimaa typically freezes completely in approximately two weeks, and the whole lake is normally covered in ice from December to beginning of May. The data was used from the period of time between the lake freezing up and the ice melting [45] or during tag drop off in the early stage of the early spring molting season (Table 1). Individuals with VHF tags (*n* = 16) were located on average three times per week (both effort and success rate) using multichannel receivers (AR 8000, AOR Ltd, and Icom IC-R20, Japan) in combination with handheld, three-element, directional antennae (ATS). The VHF fixes were collected by triangulation from receivers typically traveling by snowmobiles on ice (estimated location error of 100 m [38]). The maximum bearing capacity of the VHF signal varied in the range of 3–5 km under field conditions, depending on the landscape and weather as well as whether the animal was hauled out or submerged in water. Animals hauled out in the snow lairs or other subnivean structures were estimated by a regular unbroken signal series. Due to the freshwater environment, the radio signal can be detected underwater, enabling the detection of the haulout sites situated in underwater ice cavities. Contrary to when the seal is diving, the signal remains continuous, and the signal volume remains stable when the seal is hauled out. Individuals with GPS-phone tags (*n* = 7) were tracked with a location interval set at 20–30 min and a GSM call interval of 12 hours. The GPS-phone tags also provided data on each haul out and dive. Individuals with GPS-Argos tags (*n* = 2) were tracked with a GPS location interval set at 20 min with a maximum of six fast-GPSs per day (max 12 attempts) and a transmit control daily allowance of 110. In addition to the GPS locations (accuracy of 25–70 m [46]), the Argos class three locations with an estimated error of <250 m were utilized. The haul out locations could be separated from the underwater locations based on the tag wet-dry sensors.

The breeding success and population size of the Saimaa ringed seal are currently estimated based on the annual lair censuses. The observed haul out sites of the tracked seals in this study were investigated during these censuses in April (see [37]). Each observed location within the home range of the tagged individuals was examined, and the structure was classified as a birth lair if there were signs of pup (lanugo hair, dead pup or placenta). Other locations (with or without lair structures) were classified as haul outs.

To estimate the breeding density of the ringed seal females, the location data on lairs of non-tagged parturient females (*n* = 59) in study area were used. Pinniped females do not consume placentas after birth [47, 48]; thus, the presence of a placenta can be used as confirmation of pup birth in a specific location. Therefore, the exact birth locations in the main study area were confirmed, based on the finding of placentas and dead pups collected in underwater surveys in years 2011–2013 [39, 49]. During the study years, 46% of the birth lairs classified by lanugo hair could be confirmed based on the finding of a placenta [39].

Home range estimates can indicate areas and habitats for various behavior patterns. It is widely recognized that 100% home ranges reveal the entire feeding and excursion area of an individual, while 50% of the home range represents a smaller core areas(s) where most of the activities concentrate (e.g., [50]). Winter telemetry data was characterized by low numbers of fixes. This was especially seen in GPS-phone tag records from ice-covered season (on average 2.8 location / day, Table 1) compared to data gathered during the open-water season (18 location / day; see [21]). In addition, most of the locations were clustered around haul-out sites (S1 Table). We used the basic minimum convex polygon (MCP) method, which indicates the maximum area used, to describe the total winter time home ranges (100%) and core areas (50%) of the tracked seals using the AniMove plugin 1.4.2 in the open source QGIS (Quantum GIS Development Team, version 2.14.18, 2016, USA). The MCP method was chosen because it delineates the range using the outermost location points and does not account for the density of the recorded locations [51]. More advanced techniques, such as Local Convex Hull (LoCoH) models, do not perform well on small datasets [52] and therefore MCP 100% approach was likely more appropriate for conservation purpose, which was the main focus of this study.

The overlap of home ranges was explored between the individuals from neighboring home ranges. As the Saimaa ringed seal is known for its high degree of site fidelity (e.g., [24]), we assumed that the individual seals use the same home ranges year to year; therefore, neighbors could be investigated even when tracked in different study years. Furthermore, a recent photo-identification study (see [44]) confirmed that all neighboring seals were alive throughout the study period. It may be possible that there were other non-tagged seals in the same areas; therefore, the placenta findings were examined with respect to the breeding density independent from the instrumentation. The percent of spatial overlap of the interannual home ranges of four individuals were calculated according to Bernstein et al. [53]. To investigate the breeding density and female wintertime home ranges, the mean core area radius of the tracked females was used (buffered) to create an area around the confirmed birth lairs (placenta data). We conducted all the map analyses by using QGIS and its plugins.

**Fig 1:**
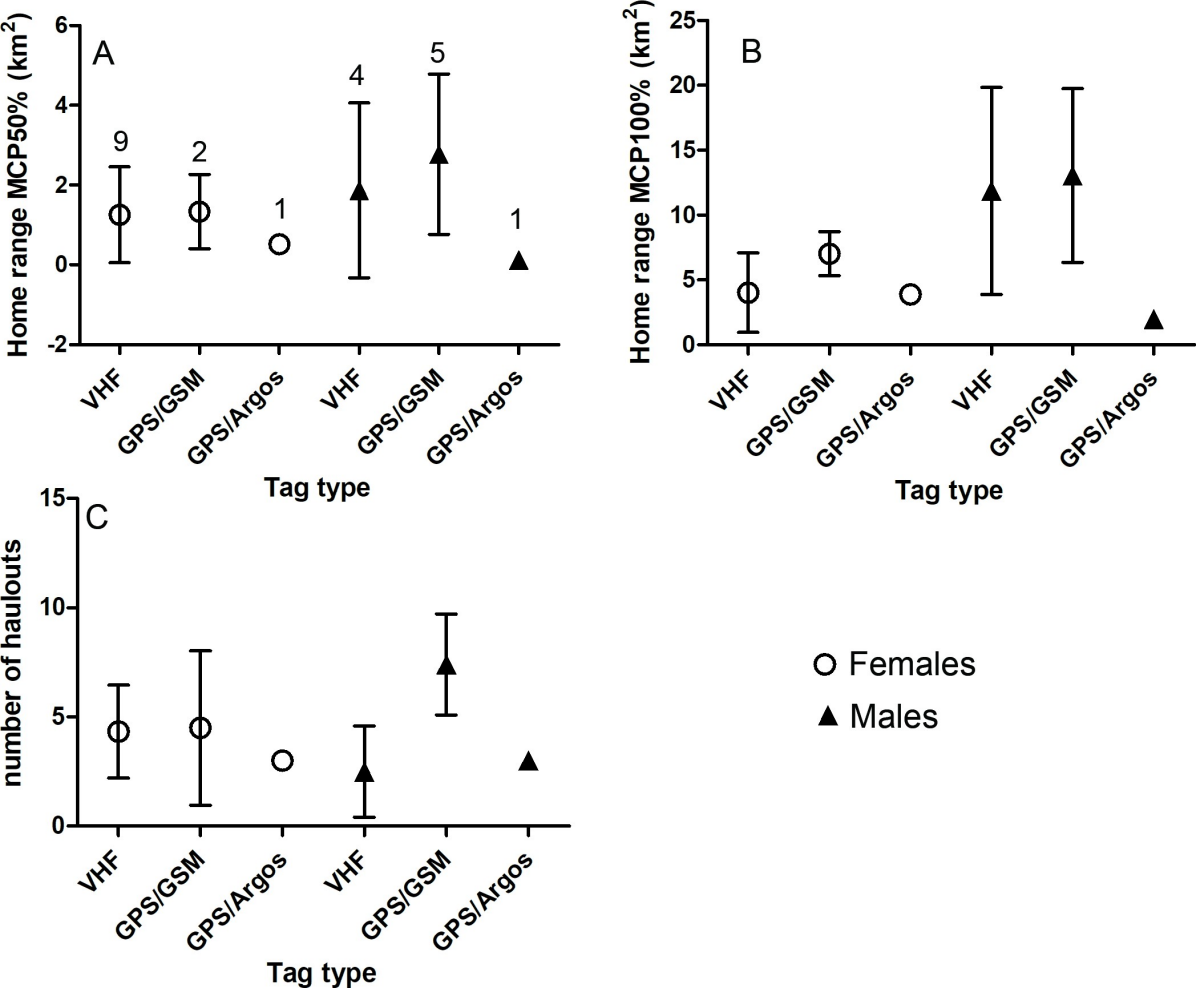
A-B) Sizes of the home ranges (km^2^) and C) number of the haul outs of the adult male and female Saimaa ringed seals tracked with different tag types. VHF = very high frequency radio tags, and GPS/GSM and GPS/Argos = satellite-based tags. The number of animals tracked with each tag is shown above the plots in Figure A.

We examined the possible effects of the number of fixes, tracking duration and sex on the home range size (Log10 transformed for normal distribution) and the number of haul outs using generalized linear mixed models (GLMM) in IBM SPSS statistics 23. Only the data of the adults were taken into account in the statistical analyses. To correct the pseudoreplication of the same individuals tracked over several seasons, we used seal identity as a random effect. The interactions of the effects could not be fitted to the home range analyses in SPSS. Furthermore, the general linear model (GLM) was used to test the sex differences in the mean haul out distances. Due to the small sample size, it was not possible to test statistically the effect of tag type. However, there was no indication that the tag type affected the results (see further Fig 1). As there was high variation in the number of fixes between datasets, we plotted the number of fixes and home range sizes for each individual to investigate the accuracy of the home range estimates, which were subsequently considered to be reliable (S1 Fig).

## Results

**Fig 2:**
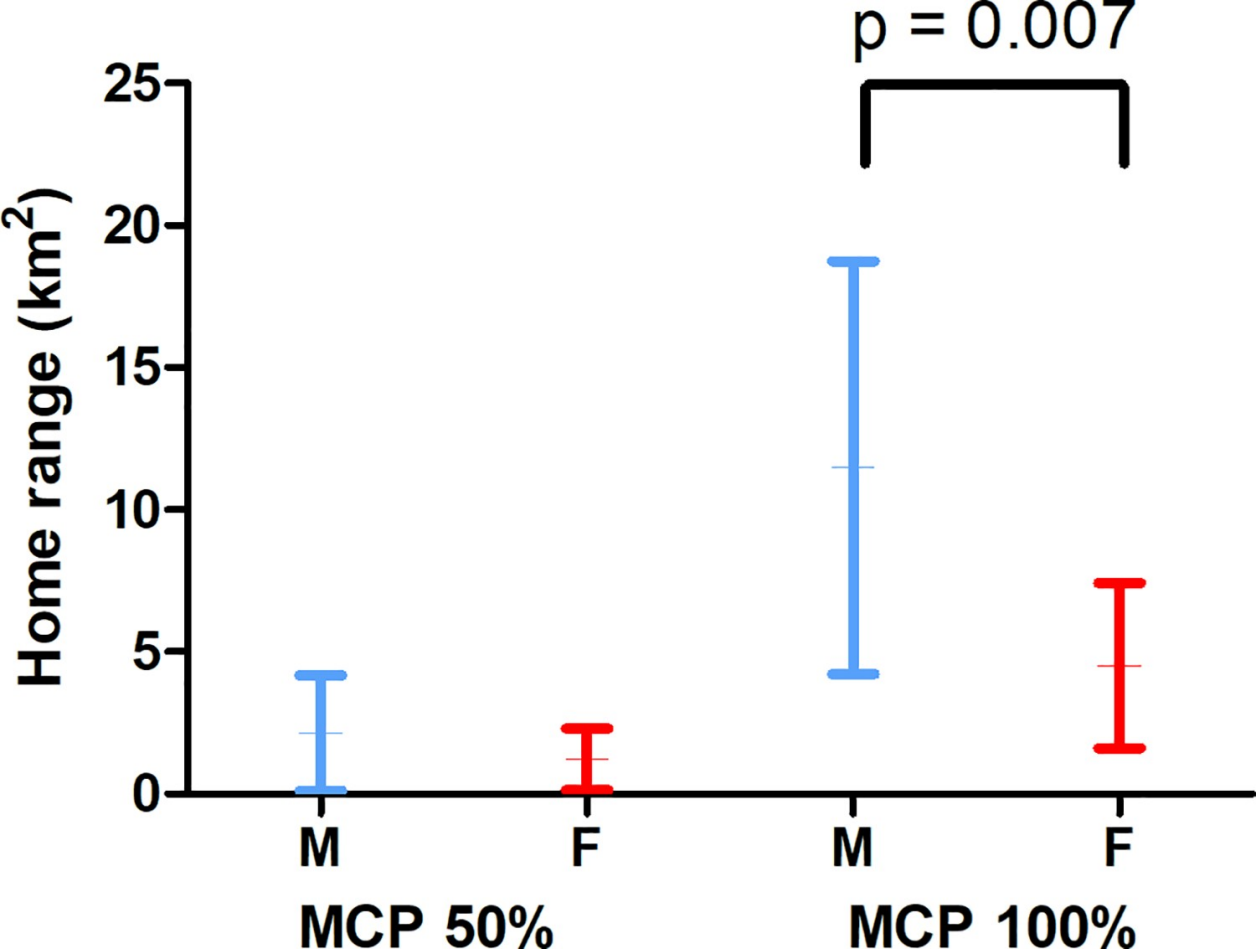
Average ± SD home range sizes (MCP50% and 100%) of the Saimaa ringed seals. Adult males (M) had statistically larger total home ranges (100%) than those of the females (F), but the core area sizes (MCP50%) were similar.

The mean home range size of all tagged seals (MCP100% ± SD) was 7.4 ± 6.1 km^2^ (Table 2, S1 Table). The number of fixes and tracking duration (GLMM, F_(1, 18),_ p > 0.05, S2 Table) did not affect the estimated adults home range sizes. However, the mean total home range sizes of the adult males were significantly larger than those of the adult females (Table 2 and Fig 2, GLMM, F_(1, 18)_ = 9.255_,_ p = 0.007, S2 Table). The core area (50%) of the adult seals (1.6 ± 1.6 km^2^) was similar in size for both sexes (Table 2 and Fig 2, GLMM: F_(1, 18)_ = 0.693_,_ p = 0.416, S2 Table) and there was no apparent differences between sexes in the effect of different tracking methods (VHF vs. GPS-based; Fig 1).

**Fig 3:**
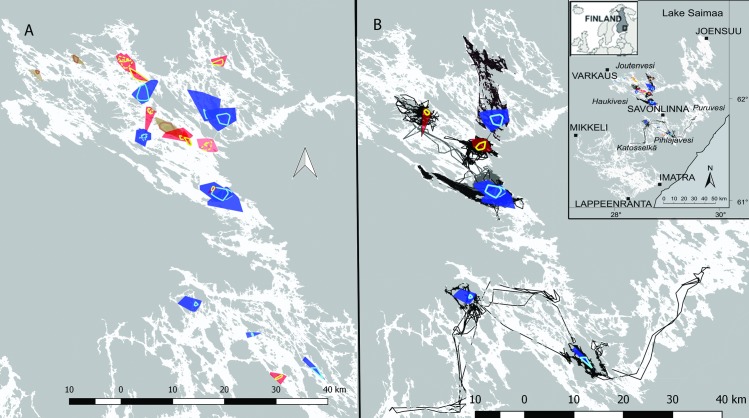
A) Winter home ranges of the tagged Saimaa ringed seals (*n* = 20; red = adult females, blue = adult males and brown = subadult polygons, MCP100%) and core areas (yellow = females, light blue = males and light brown = subadult line polygons, MCP50%). The core area lines of the seals with multiple interannual tracking seasons are dotted. B) Winter home ranges and open-water tracks (black and gray lines) of the GPS-GSM tagged Saimaa ringed seals (*n* = 7, MCP100%). Basemap (C) Land survey of Finland 5/2017.

The study period of the individual seals ranged from 26 to 126 days and covered in majority of the cases part of the main breeding season in February–March (Table 1). On average, we obtained one location per animal per day (VHF 0.3, GPS-GSM 2.8 and GPS-Argos 0.9). There was evidence of parturition in three of the tagged females. In the first case, the birth of a live pup was confirmed during annual lair census. In the second case, the pup was stillborn, and, in the third case, the pup was nursed on the ice after the female abandoned a lair that collapsed due to poor snow conditions. The animals moved in smaller areas during the wintertime than during the open-water season (Fig 3B). Two subadult males overwintered in the edges of the study area (Fig 3A).

Annually and interannually, the tagged individual females’ core areas did not overlap with each other. In one instance, a male core area overlapped with both another male and a female seal (Fig 3). The home ranges of the four individuals with multiple interannual tracking seasons overlapped on an average of 28% (range 13–56%) (MCP100; 31%, MCP50; 27%). One exception was a male (UR99, Table 1) that had three different tracking seasons. Home range center point of subadulthood differed 24 km from those of adulthood (not included in the average). The center points of each of the adult (*n* = 4) interannual home ranges and core areas (MCP100 and MCP50) were an average of 800 ± 420 SD m and 550 ± 330 m apart from each other, respectively.

**Fig 4:**
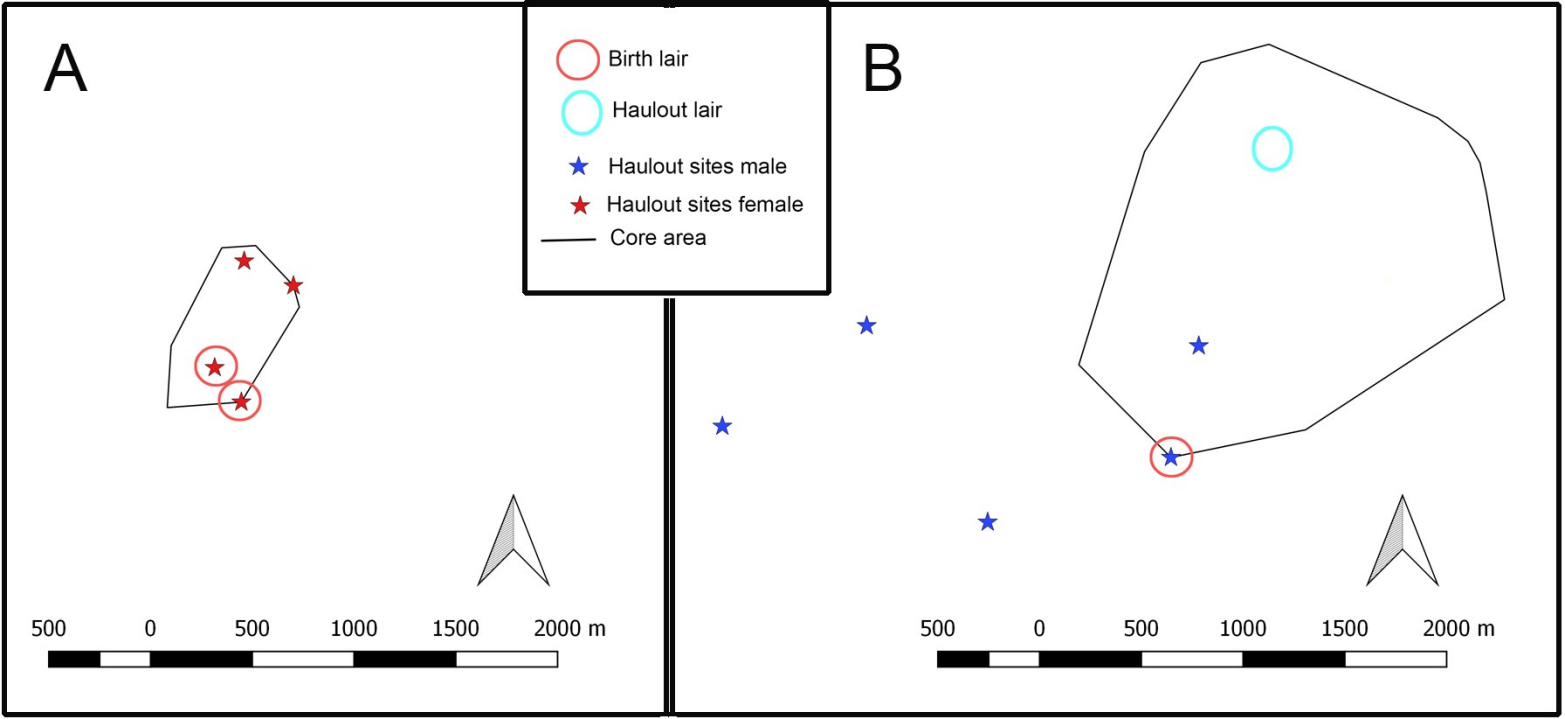
**An example of A) tagged female and B) tagged male Saimaa ringed seal haul outs and the lairs found in their core areas.** The map underneath the sites has been removed to protect the confidentiality of the exact lair sites of the Saimaa ringed seals.

The average number of haul out sites (lairs or other subnivean structures) per tagged seal was 4.5, but there was a large individual variation in the site numbers (range 0–10; Table 2, S1 Table and Fig 4). The sex, number of the fixes, the tracking duration or their interactions did not effect to the number of the haul outs statistically (GLMM, Poisson-fitted, S2 Table). The mean ± SD distance between the haul out sites of the individual seals was 1.6 ± 1.1 km (range 0.2–5.9 km). The haul out sites (*n* = 50) individually used by adult males were significantly (GLM, F_(1)_ = 60.706 p = 0.000) further apart from each other (mean ± SD: 1.9 ± 1.3 km, range 0.2–5.9 km) compared with those (*n* = 51) used by the adult females (1.2°± 0.7 km, range 0.2–3.0 km). Overall, the haul out sites of individual seals were situated an average ± SD of 1.7 ± 2.1 km^2^ inside their home ranges (adult males 2.7 ± 2.9 km^2^, adult females 1.0 ± 0.9 km^2^).

During the annual lair census, only 30% of the overall haul out sites detected with telemetry were confirmed to have visible lair structures (Table 2). Almost half of the tracked adult female haul out sites (43%) showed lair structures compared with only 14% of the male haul out site. Most of the tracked seals, both in visible lairs and other haul outs, were situated inside of their core areas (Table 2 and Fig 4). The core areas of four tagged males also contained birth lairs, but the core areas of both tagged and non-tagged females did not contain other individual’s birth lairs. Before the pupping season, two tagged males occupied lairs that were used as birth lairs by females later in the season. The males moved to other lairs after January.

The haul out activities during the ice-covered season were recorded from the GPS-phone tags on seven tracked seals (S3 Table). The mean ± SD period spent hauled out was 5 h 41 min°± 2 h 36 min (range of means 1 h 34 min– 9 h 1 min, minimum 9 min, maximum 23 h). The mean ± SD duration between the haul out bouts was 1 d 18 h ± 1 d 13 h (range of means 4 h– 4 d 16 h, minimum 3 min, maximum 17 d 11 h).

According to the birth lair locations based on placental data (*n* = 59, independent from the instrumentation), the mean annual minimum distance between two lairs of parturient females was 3.9 km (range 0.8–16.3 km) in the study area. In addition, the buffer zones with a radius of 621 m (corresponding to the core area sizes of the tagged females) around birth lairs did not overlap with other birth lair locations.

## Discussion

Saimaa ringed seals constrict their movements during the ice-covered season in winter, when the home range (approximately 7 km^2^) was 13 times smaller than that in summer (approximately 90 km^2^) [8, 21]. Constricted movements during the breeding season are also seen in marine ringed seals [12–14, 17]. The adult males occupy larger areas (see also [16]), and the home ranges were twice the size of those of the females. However, the most intensively used core areas (1.6 km^2^) were similar in size with both sexes, and they were three times smaller during the winter compared to those in the summer [8, 21]. In the tagged seals, most of the haul out sites and all the birth lair sites were within the core areas, where the seals’ main activity was concentrated throughout the study period. In addition to haul out, the use of core areas is linked to breeding and nursing. Female-pup pairs spend large amounts of time in the vicinity of the natal site until weaning [54], and the average total home range size of the pups is some 2.0 km^2^ during the nursing season [38]. Ringed seals are suggested to exhibit interannual breeding time site fidelity [22, 55], and this was confirmed in our study, as the adult seals stayed in the same regions during two different breeding seasons; the core areas center points of the interannual home ranges were within around half kilometer.

We suggest that adult ringed seal females exhibit avoidance during the breeding season, which may indicate some degree of territoriality. This suggestion was supported by our findings of a lack of core area overlap between the adult females. During the breeding season, ringed seal females may defend the most intensively used home range areas against other females, or they may simply avoid other females. Smith and Hammill [22] reported the possibility that parturient female ringed seals maintain territories around potential birth lair sites, and our study provides the strongest evidence on ringed seal female territoriality to date. The lack of a core area overlap between females when related to reproduction has been reported in many small [56–59] and some medium-sized mammals [60, 61]. Furthermore, some female primates have been found to be more aggressive inside their core areas and avoid other females [62, 63]. The function of female territoriality during the breeding season has been assumed to be related to resource defense [64]. It is well known that Saimaa ringed seals exhibit high site fidelity [4, 19, 20, 44] and that females are especially philopatric [65]. Therefore, we suggest that a lair site with a sufficient annual snow cover for birth lair creation could be a resource worth of interannual defense by the ringed seal females.

During the mating season, slightly polygynous ringed seal males [25, 66] are known to defend underwater territories against other males around the lairs occupied by one or several females [13, 22, 55, 67]. The evidence of males occupying female home ranges was also observed in our data; four tagged males had females (i.e., there were birth lairs) in their core areas. In addition, on two occasions, males occupied a lair during early winter, and a female used the same lair for pupping later in the breeding season. This finding is in accordance with the observations of Kelly et al. [17] of adult males sharing breathing holes with adult females during the breeding season. Therefore, we suggest that, during the breeding season, the male mating strategy may involve not only guarding the female but also providing resources (lairs). There is evidence of haul out lair sharing among the ringed seals in Lake Ladoga and in the Arctic, which has been suggested to be related to the social behavior of the Ladoga ringed seals and the Arctic subadults rather than a mating tactic [1, 5]. In our data, there was not such evidence on lair sharing.

Based on the findings in the Arctic, the adult ringed seals occupy shore-fast ice areas for breeding, while subadults stay at the edge areas where the ice conditions are not as stable (e.g., [13, 25]). Similar evidence was found in our study in Lake Saimaa, where two subadult males inhabited the edges of the breeding areas. One of these males was found seven years later as an adult occupying a home range in the middle of the breeding area, where it stayed during two consecutive tracking periods. This finding on subadults using the edge areas is notable and highlights the importance of conservation awareness, not only in the main breeding areas but also on the edges of distribution areas, which may be especially important for the subadults. Protecting the edge areas ensures not only the next breeding cohort but also the long-term recolonization of lake regions from where Saimaa ringed seals disappeared during the mid-20 century.

This study emphasized the importance of wintertime resting locations for the conservation of Saimaa ringed seals. The ringed seals spent an average of almost six hours during every second day hauled out on subnivean snow/ice structures, which is similar to duration of the haul out patterns in the summer [20]. Most conservation efforts have been typically focused on the seal birth sites on Lake Saimaa (e.g., [21, 37, 40, 54]), but our findings also highlight the conservation needs of winter time haul out sites in general.

Our results have implications for the current population size estimation method based on the lair censuses, especially when the individual Saimaa ringed seals may use more subnivean structures during winter than previously considered. It was assumed that ringed seals use one to three lairs during the winter [23]; however, in this study, the seals occupied an average 4.5 haul out sites. Kelly and Quakenbush [16] observed an average use of two to four lairs by radio tagging ringed seals in the Alaskan Arctic. The haul out locations of the Saimaa ringed seals were situated at a distance of 1.6 km, which is relative long distance taking account of restricted wintertime movements. Therefore, not only the number of lairs, but also distance between them forms challenges for reliability on population size estimate based on lairs. Notably, not all seals had visible lairs during the censuses in April, and one animal could have 10 different haul out sites. In total, only a third of the haul outs were found to be snow lairs. Some of the lairs may have collapsed and melted before the census; furthermore, some individuals may have dug new lairs after the tag drop off. In addition, ringed seals use not only lairs but also other subnivean structures (such as under ice air spaces) for resting. Therefore, we suggest that the population estimates relying only on lair numbers and distribution [37] may not provide an accurate numbers of individuals, especially in mild winters. We suggest that lairs should be handled with caution as an index of animal abundance due to the large individual variation in the number of lairs used and the increasing negative effects of mild winters on the visibility of subnivean lair structures during the censuses. Additional and alternative census methods, such as mark-recapture approaches based on photo-identification [44], should be taken into consideration for the safeguarding of accurate population estimates, particularly during varied climate conditions.

The effective long-term conservation of the Saimaa ringed seal population must not only ensure the protection of the current favorable breeding areas but also predict future habitat needs. Although the seal population has slowly increased during the last decade [68], the current population, at less than 400 seals, is far from the potential population level. When calculating the theoretical carrying capacity of Lake Saimaa by regarding the core areas and accounting for the avoidance of females, and the fact that already almost third of the lake area is no longer suitable for seal lair sites due to intensive land usage [40], over 4 000 Saimaa ringed seals (sex ratio 1:1 [37]) could occupy the lake. According to Liukkonen et al. [40], the Saimaa ringed seals lairs are currently located closer to the sources of anthropogenic disturbances than they were in earlier times, and the land use intensity has a negative effect on neonate survival. Therefore, land usage planning plays a major role in continuing the positive growth trend of the population. We suggest that the effectiveness and transparency of decisions made regarding conservation would benefit from knowledge of the spatial ecology of seals. The core area sizes of all tagged seals (1.7 km^2^) found in this study, which equates ca. 700 m radius, supports the discovery of 800 m buffer zones in studies on the home range size of the nursed pup [21] and the link between human induced disturbances and an increased perinatal pup mortality [40]. The minimum magnitude of the buffer zones around birth lairs should be taken into account during land usage planning to mitigate the effects of disturbances caused by humans.

The knowledge of the spatial patterns and home ranges of endangered species is a useful tool on which to base in situ conservation measures and to implement sustainable land use planning in key habitats and seasons. Tracking individuals throughout the ice-covered seasons provided insights into the movement ecology of the Saimaa ringed seal during the winter, which is the key element related to the conservation. Climate change forms a major challenge for the future of this endangered population. The ice thickness and the snow cover of the subnivean lair have direct implications for the survival of ringed seal pups. Changing climate conditions with cumulative effects of varied anthropogenic factors (e.g., [40]) may have drastic influences on the breeding habitats and long-term survival of the population. Seals may be able to cope with the changing environments due to behavioral plasticity, such as the timing of reproduction and a shortened nursing period [13, 26, 69, 70]. However, due to the restricted lake habitat and small population size, rapid adaptation response to environmental change may include negative consequences. Therefore, active and unprejudiced conservation strategies should be emphasized to mitigate the most acute threats that can be impacted, such as bycatch mortality and habitat destruction via the industrial development of the lake. Our results on interannual site fidelity, together with the documentation of restricted movement, further highlight the importance of breeding time habitats as a central focus for conservation measures. In addition, our results underline the importance of the immediate surroundings of lair sites. Therefore, based on this study and two earlier works [21, 40], we urgently recommend that environmental authorities look more closely at the role of human-disturbance-free buffer zones in breeding areas and effectively implement those zones into the land usage planning practices of the Lake Saimaa region.

## Supporting information

S1 Table Description of the haul out and home range sizes of the tagged Saimaa ringed seals. HO = haulout, S = submerge, HR = home range.
(DOCX)

S2 Table Description of the statistical analyses regarding the GLMM model. Statistically significant variables are highlighted. A) Normal fitted GLMM model for the lg10 transformed total home range (MCP100), ID as a random effect, AIC = 40.597, B) Normal fitted GLMM model for the lg10 transformed core area home range (MCP50), ID as random effect, AIC = 58,082. C) Full Poisson-fitted GLMM model for number of haul out sites, ID as a random effect, AIC = 124.405. D) Final Poisson-fitted GLMM model for the number of haul out sites, ID as a random effect, AIC = 116.231.
(DOCX)

S3 Table Description of the haul out activities of seven GPS-phone tagged Saimaa ringed seals during winter. (DOCX)

S1 Fig **Discovery curves (home range size km^2^ vs. number of fixes) of A) the VHF-radio tagged and B) the GPS-tagged Saimaa ringed seals**.
(TIF)
