# Supplementary material for: Winter behavior of Saimaa ringed seals: Non-overlapping core areas as indicators of avoidance in breeding females
Source: PLoS One. 2019 Jan 4;14(1):e0210266. doi: 10.1371/journal.pone.0210266 (PMC6319809; doi:10.1371/journal.pone.0210266)
Supplement: S1 Table — HO = haulout, S = submerge, HR = home range. (DOCX) [file pone.0210266.s001.docx]

**S1 Table. Description of haul out and home range sizes of tagged Saimaa ringed seals.** HO = haul out, S = submerge, HR = home range.

| **Seal**  **ID** | ***n* fixes**  **HO** | ***n* fixes S** | ***n* HO** | **Mean ± SD distance between HO (km)** | **HR km^2^**  **(MCP100)** | **Core area km^2^**  **(MCP50)** |
| --- | --- | --- | --- | --- | --- | --- |
| MI99 | 17 | 17 | 1 | **-** | 3.7 | 3.7 |
| UR99 | 1 | 17 | 1 | - | 2.9 | 0.3 |
|  | 8 | 35 | 5 | 1.2 ± 0.5 | 5.3 | 1.0 |
|  | 4 | 24 | 3 | 0.8 **±** 0.2 | 6.6 | 0.6 |
| EL06 | 18 | 13 | 5 | 1.4 **±** 0.9 | 2.5 | 0.9 |
| HE07 | 12 | 25 | 9 | 1.5 **±** 0.7 | 8.4 | 2.5 |
|  | 20 | 19 | 4 | 1.4 **±** 0.7 | 3.1 | 1.5 |
| KJ07 | 0 | 20 | 0 | - | 22.8 | 0.8 |
| TO07 | 22 | 179 | 9 | 2.1 ± 1.1 | 14.6 | 5.3 |
| PA09 | 4 | 12 | 3 | 0.6 **±** 0.1 | 2.1 | 0.9 |
| KA10 | 13 | 14 | 4 | 0.5 **±** 0.2 | 1.0 | 0.3 |
| NO10 | 14 | 46 | 7 | 0.7 ± 0.3 | 10.5 | 4.6 |
| OL10 | 607 | 31 | 7 | 0.9 **±** 0.4 | 5.8 | 0.7 |
|  | 6 | 31 | 3 | 0.7 ± 0.3 | 1.4 | 0.3 |
| LI10 | 6 | 48 | 5 | 0.9 **±** 0.4 | 9.8 | 0.4 |
| TE07 | 15 | 23 | 4 | 1.0 **±** 0.4 | 2.1 | 0.2 |
|  | 2 | 120 | 2 | 0.3 **±** 0 | 8.2 | 2.0 |
| AS12 | 9 | 230 | 5 | 1.4 **±** 0.6 | 19.1 | 2.9 |
| VO12 | 34 | 49 | 10 | 2.4 **±** 1.2 | 19.2 | 4.0 |
| HH12 | 4 | 21 | 4 | 1.2 **±** 0.5 | 4.3 | 1.5 |
| NI09 | 3 | 35 | 2 | 0.2 ± 0 | 12.8 | 5.2 |
| MI13 | 58 | 49 | 8 | 1.0 **±** 0.5 | 8.5 | 0.3 |
| JE14 | 19 | 11 | 5 | 3.4 ±1.9 | 4.0 | 1.4 |
| PA16 | 10 | 12 | 3 | 1.4 **±** 0.7 | 2.0 | 0.1 |
| PI16 | 19 | 36 | 3 | 1.8 **±** 0.4 | 3.9 | 0.5 |
