# Supplementary material for: Winter behavior of Saimaa ringed seals: Non-overlapping core areas as indicators of avoidance in breeding females
Source: PLoS One. 2019 Jan 4;14(1):e0210266. doi: 10.1371/journal.pone.0210266 (PMC6319809; doi:10.1371/journal.pone.0210266)
Supplement: S2 Table — Statistically significant variables are highlighted. A) Normal fitted GLMM model for the lg10 transformed total home range (MCP100), ID as a random effect, AIC = 40.597, B) Normal fitted GLMM model for the lg10 transformed core area home range (MCP50), ID as random effect, AIC = 58,082. C) Full Poisson-fitted GLMM model for number of haul out sites, ID as a random effect, AIC = 124.405. D) Final Poisson-fitted GLMM model for the number of haul out sites, ID as a random effect, AIC = 116.231. (DOCX) [file pone.0210266.s002.docx]

**S2 Table. Description of the statistical analyses regarding the GLMM model.** Statistically significant variables are highlighted. **A) Normal fitted GLMM model for the lg10 transformed total home range (MCP100)**, ID as a random effect, AIC = 40.597, **B) Normal fitted GLMM model for the lg10 transformed core area home range (MCP50)**, ID as random effect, AIC = 58,082**. C) Full Poisson-fitted GLMM model for number of haul out sites**, ID as a random effect, AIC = 124.405. **D) Final Poisson-fitted GLMM model for the number of haul out sites**, ID as a random effect, AIC = 116.231.

A

| **Effect** | **F_(1,18)_** | **p** |
| --- | --- | --- |
| gender | 9,255 | 0,007 |
| number of fixes | 1,540 | 0,231 |
| number of tracking days | 2,612 | 0,123 |

B

| **Effect** | **F_(1,18)_** | **p** |
| --- | --- | --- |
| gender | 0,693 | 0,416 |
| number of fixes | 0,203 | 0,657 |
| number of tracking days | 0,440 | 0,516 |

C

| **Effect** | **F _(1,18)_** | **p** |
| --- | --- | --- |
| gender | 0,000 | 0,995 |
| number of fixes | 5,835 | 0,028 |
| number of tracking days | 0,158 | 0,696 |
| gender*fixes | 3,353 | 0,086 |
| gender* tracking days | 0,264 | 0,615 |

D

| **Effect** | **F_(1,18)_** | **p** |
| --- | --- | --- |
| gender | 0,046 | 0,833 |
| number of fixes | 2,732 | 0,116 |
| number of tracking days | 0,063 | 0,805 |
