# Supplementary material for: Winter behavior of Saimaa ringed seals: Non-overlapping core areas as indicators of avoidance in breeding females
Source: PLoS One. 2019 Jan 4;14(1):e0210266. doi: 10.1371/journal.pone.0210266 (PMC6319809; doi:10.1371/journal.pone.0210266)
Supplement: S3 Table — (DOCX) [file pone.0210266.s003.docx]

**S3 Table. Description of the haul out activities of seven GPS-phone tagged Saimaa ringed seals during winter.**

| **Seal ID** | **Number of haul out events** | **Mean ± SD haul out duration (hh:min:ss)** | **Min-max haul out duration**  **(hh:min:ss)** | **Mean ± SD duration between haul outs**  **(hh:min:ss)** | **Min-max duration between haul outs**  **(hh:min:ss)** |
| --- | --- | --- | --- | --- | --- |
| TO07_0910 | 37 | 7:10:14 ± 3:56:25 | 0:16:30-14:05:15 | 58:59:50 ± 52:30:47 | 0:03:45-244:02:45 |
| OL1011 | 159 | 7:24:03 ± 6:34:43 | 0:09:00-28:23:15 | 11:27:23 ± 17:23:35 | 0:03:15-124:35:30 |
| TE07_1112 | 7 | 9:01:28 ± 5:22:50 | 0:12:00-15:33:30 | 112:14:20 ± 126:21:03 | 0:03:45-348:41:00 |
| AS12 | 13 | 6:31:29 ± 3:01:57 | 2:48:12-12:20:12 | 59:26:58 ± 39:58:18 | 17:44:48-145:39:24 |
| VO12 | 473 | 1:34:28 ± 2:32:27 | 0:10:16-16:52:40 | 4:23:29 ± 23:27:50 | 0:03:04-419:15:04 |
| MI13 | 53 | 4:53:07± 3:00:39 | 0:14:08-12:16:00 | 30:10:06 ± 28:55:59 | 0:03:24-111:57:12 |
| JE14 | 36 | 3:14:40 ± 4:32:13 | 0:10:40-14:05:52 | 17:55:20 ± 42:22:53 | 0:09:00-206:45:32 |
