# Supplementary figures and images for: Winter behavior of Saimaa ringed seals: Non-overlapping core areas as indicators of avoidance in breeding females
Source: PLoS One. 2019 Jan 4;14(1):e0210266. doi: 10.1371/journal.pone.0210266 (PMC6319809; doi:10.1371/journal.pone.0210266)

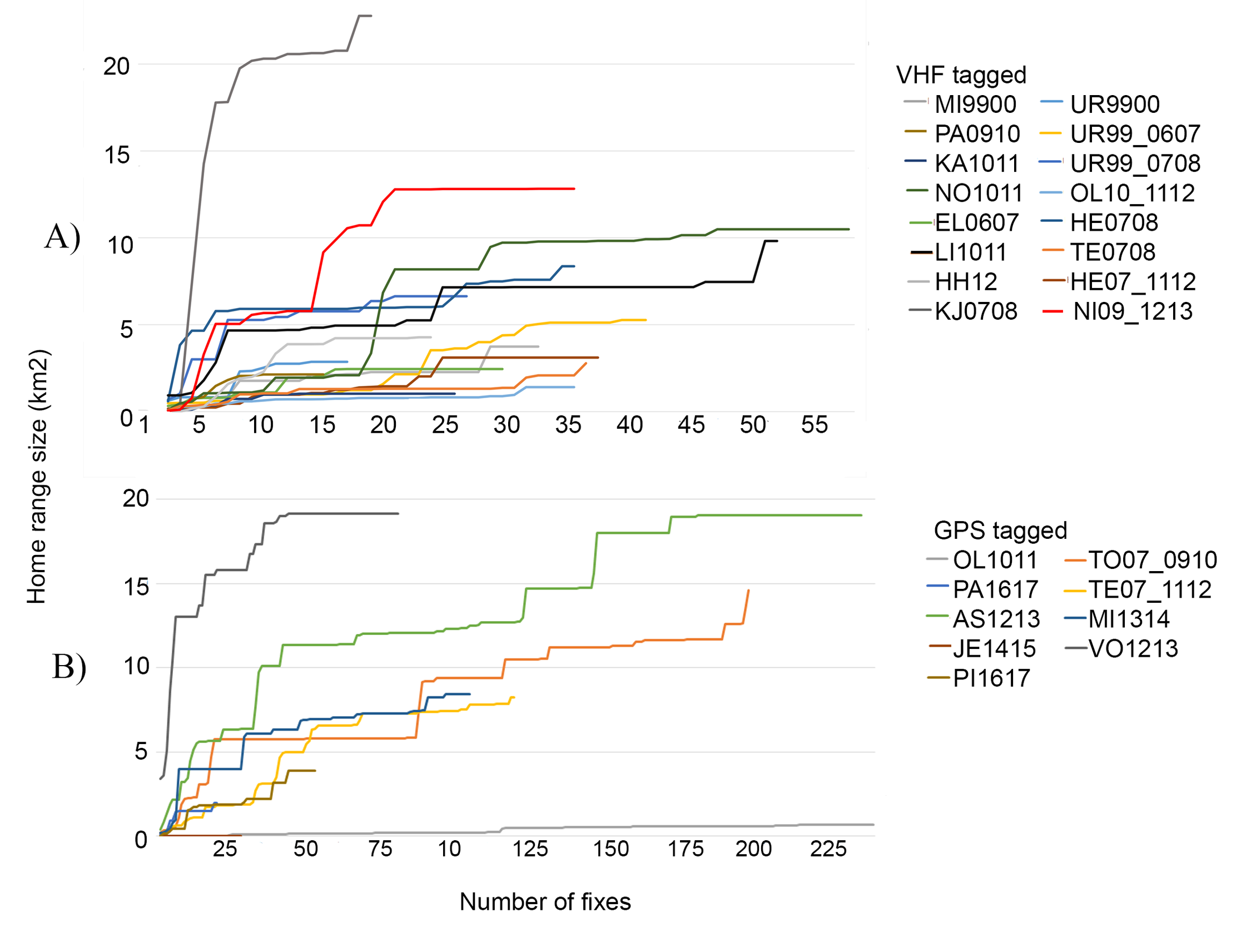

Supplement: S1 Fig — Discovery curves (home range size km2 vs. number of fixes) of A) the VHF-radio tagged and B) the GPS-tagged Saimaa ringed seals. (TIF) [file pone.0210266.s004.tif]
